# ABO blood group antigen therapy: a potential new strategy against solid tumors

**DOI:** 10.1038/s41598-021-95794-x

**Published:** 2021-08-10

**Authors:** Qiong Luo, Mingxin Pan, Hao Feng, Lei Wang

**Affiliations:** 1 Department of General Surgery, Affiliated Hengyang Hospital, Southern Medical University (Hengyang Central Hospital), Hengyang, 421000 China; 2 General Surgery Center, Department of Hepatobiliary Surgery II, Guangdong Provincial Research Center for Artificial Organ and Tissue Engineering, Guangzhou Clinical Research and Transformation Center for Artificial Liver, Institute of Regenerative Medicine, Zhujiang Hospital, Southern Medical University, Guangzhou, Guangdong Province China; 3 National Cancer Center/National Clinical Research Center for Cancer/Cancer Hospital, Chinese Academy of Medical Sciences and Peking Union Medical College, Beijing, 1000210 China; 4 Medical Laboratory Center, Chifeng Municipal Hospital/Chifeng Clinical College, Inner Mongolia Medical University, Chifeng, 024000 China

**Keywords:** Cancer, Immunology

## Abstract

The economic burden of tumors is increasing, so there is an urgent need to develop new therapies for their treatment. Killing tumors by activating complement is an effective strategy for the treatment. We used the ABO blood group system and the corresponding antibodies to activate the killer cell capacity of the complement system. After the construction of a mouse model containing blood group A antibodies and inoculating colorectal cancer and breast cancer cells into the axillae of the mice, intratumoural injection using a lentivirus carrying a blood group antigen as a drug significantly reduced the tumor volume of the mice. Compared with the control group, the content of the C5b-9 complement membrane attack complex in the tumors of mice treated with the blood group A antigen was significantly increased, and the proportion of NK cells was also significantly increased. In vitro cell-based experiments proved that tumor cells expressing blood group A antigens showed significantly inhibited cell proliferation when added to serum containing blood group A antibodies. These results all prove that the ABO blood group antigen may become a powerful tool for the treatment of tumors in patients.

## Introduction

Cancer is the number one cause of death in China, including various disease forms, resulting in a huge economic and spiritual burden^1^. Statistics from the World Health Organization show that surgery, radiation therapy and chemotherapy are the three main cancer treatments applied and that the resulting cure rates for cancer are very low^2^. There are still a large number of cancer patients who are not well treated, and the development of new treatments is very important for cancer patients.

Immunotherapy is a new cancer treatment method^3^.Chimeric Antigen Receptor T-cell immunotherapy (CAR-T) with chimeric antigen receptors has shown great progress as an immunotherapy method^4^, but ultimately result in off-target effects during immunotherapy^5^. Regardless of the underlying explanation on off-target effects, the reduction of tumour cell surface antigens is the most important mechanism underlying tumour cell escape^6,7^. If we change our thinking and actively express an antigen on the surface of the tumour cell membrane, the immune system can attack the tumour and reduce the number of tumour cells.

ABO blood group antigens can be widely distributed in Red Blood Cell (RBCs), platelets, white blood cells, plasma proteins, certain tissues and various cell surface enzymes found in blood group antigens^8–10^. The epithelial tissues of the oral cavity, gastrointestinal tract, lungs, bladder, breast, cervix and prostate contain ABH antigens, but malignant tumours of these tissues lack ABO blood group antigen expression^11–15^. In 1953, the relationship between the ABO blood group system and cancer was recorded for the first time^16^.Loss of blood group antigen expression has been detected in primary breast tumors and their metastases. Loss of blood group antigen expression may be considered as a marker of invasion ^17,18^. 50% of proximal colon tumors in colon cancer show a loss of antigen expression^19^. The association between blood group A and increased risk of stomach cancer is the strongest link for any of the cancers. The finding that patients with A blood type are more susceptible to H. pylori, a known stomach cancer causing agent, provides a mechanistic explanation for how a histoblood group antigen could promote tumorigenesis^20^. Despite the multitude of studies attempting to correlate ABO phenotype with cancer risk, the link between expression of histoblood group antigens and tumorigenesis and the mechanism of action was unclear for most tumor types evaluated. ABO blood group antibodies naturally exist in the human body and activate the body's immune system by recognizing blood group antigens to produce red blood cell lysis and death^21^. Similarly, ABO blood group antigens can activate human immune function and exert anti-tumor effects. If the immune effect of blood group antibodies is used in tumor treatment, a mechanism similar to that of destroying red blood cells after blood group incompatibility can achieve the purpose of destroying tumor cells. This may become a new method to treat tumors and prevent tumor resistance.

Based on these findings, we boldly speculate that blood group antigens can be expressed on the surface of human tumour cell membranes. The blood group antigens bind to the corresponding antibodies in human serum to stimulate the body's immune system to induce erythrocyte-like lysis to eliminate tumour cells and reduce the size of the tumour. Patients with blood type A choose blood group B antigens for treatment, and patients with blood type B choose blood group A antigens for treatment, by activating the body's immune system to produce and lyse tumor cells to achieve the purpose of eliminating tumors. Here, we prove through experiments that tumours expressing ABO blood group antigens can be used as a new strategy for the treatment of solid tumours.

## Materials and methods

The experimental animal welfare was conducted in accordance with the Chinese Guidelines for Animal Welfare and approved by the Laboratory Animal Ethics Committee, Zhujiang Hospital of Southern Medical University. The study was carried out in compliance with the ARRIVE guidelines2.0.

### Establishment of cell lines capable of expressing blood group antigens

MC38, CT26 and 4T1 cells were obtained from the Cell Resource Center of the Shanghai Academy of Life Sciences, Chinese Academy of Sciences. All cells were cultured in RPMI-1640 medium supplemented with 10% foetal bovine serum (Thermo Fisher, USA). All tumour cell lines were cultured in a humidified atmosphere at 37 °C with 5% CO_2_. The blood type A lentiviral vector construct and the prepared titration of the virus (1 × 10^8^ TU/ml) were donated by the laboratory of Beijing Likeli. The MC38 and 4T1 tumour cell lines were infected at an MOI of 1:5, and the cells were collected 48 h after infection to detect the mRNA expression level of the N-acetylgalactosamine transferase gene.

### Establishment of a mouse model containing the blood group A antibody

Six to eight week old female C57BL/6J and Babl/c mice were purchased from the Beijing Huafukang Company. Under SPF conditions, the mice were housed in cages with a maximum of five mice per cage in the Experimental Animal Centre of Southern Medical University. For three consecutive weeks, an A-type red blood cell suspension from Shanghai Blood Biomedical Co., Ltd. (China) was diluted to obtain a 5% red blood cell suspension 200 μL volume was intraperitoneally injected into the mice once a week. In the fourth week, the mice were sacrificedwhole blood was collected from the mice using the eyeball removal method, and their peripheral serum was collected.

### Identification of antibodies

Whole blood was collected from the mice using the eyeball removal method. After the blood coagulated, the serum was collected by centrifugation at 1000 rpm for 10 min. A drop of the commercial A-type red blood cell suspension from Shanghai Blood Biomedical Co., Ltd. (China) was added to the test paper for blood group identification, followed by the addition of a drop of mouse serum, gentle mixing, and observation for agglutination after 2–5 min. One to two drops of standard blood were added to a small test tube, followed by the addition of a drop of mouse serum, mixing it at 1500 rpm, centrifugation for 1 min, removal of the test tube, flicking the bottom of the tube to cause the precipitate to bounce, and observation of the result under good light. If the red blood cells have agglutinated significantly, it means that the serum contains the corresponding blood group antibody and the mouse model is successfully constructed.

### Macroscopic observation of cell proliferation

Colorectal cancer cell lines MC38 and CT26 cells were selected, and the breast cancer cell line 4T1 was spread in 24-well plates at a density of 5 × 10^4^ cells per well. The MOI was 1:5, after infection with the lentivirus carrying the ABO antigens for 24 h, the fluid was changed. The medium was configured according to a mouse serum: foetal bovine serum ratio = 1:1 and added to a 24-well plate, and the results were observed after 3 days. Use an inverted microscope to observe the cell status and take pictures.

### CCK8 assay of cell proliferation

Lentiviruses carrying ABO blood group antigens were infected into the CT26, MC38 and 4T1 tumour cell lines at an MOI of 1:5. After 2 days, the cells were digested and 1 × 10^4^ cells were plated into 96-well cell culture plates. The fluid was changed. The medium was configured according to a mouse serum: foetal bovine serum ratio = 1:1. Ten microlitres of the CCK8 (Dojindo, Japan) detection reagent was added to each well at different times (4 h, 12 h, 24 h, 36 h), and the absorbance at 450 nm was measured at 2 h. The absorbance was measured at 450 nm, and a curve was drawn to assess the number of cells. The higher the absorbance value, the more tumor cells that survived.

### Reverse transcription-quantitative polymerase chain reaction (RT-qPCR)

The RNA extraction kit manufactured by Tiangen Biochemical Technology Co., Ltd. (Beijing) was used to extract total RNA according to the manufacturer’sinstructions. Total RNA was reverse transcribed into cDNA using an RR036A reverse transcription kit (TAKARA, Japan). cDNA was amplified using an RR820A qPCR kit (TAKARA, Japan). After PCR amplification, data analysis was performed using Bio-Rad IQ5 software, and GAPDH was used as the internal reference gene. The relative quantitative 2^- △△Ct^ method was used to represent the data, and all experiments were performed in triplicate. The following target-specific primers were used (all obtained from Eurofins):

- GAPDH for 5′–TGTGGGCATCAATGGATTTGG–3 ′,
- GAPDH rev 5′–ACACCATGTATTCCGGGTCAAT–3 ′,
- *N*-acetylgalactosamine transferase for 5′–TCCTCTGTGGATCAGCAATAGG–3 ′,
- *N*-acetylgalactosamine transferase rev 5′–TTAGGCTGGGTGTCAACCTTT–3 ′.

### Tissue preparation and immunohistochemistry

Tumour tissues were harvested from euthanized mice and cut in half using ophthalmic scissors. Perform routine operations according to immunohistochemistry procedures. Tissue sections were then incubated with a primary antibody against the C5b-9 complement membrane complex (Abcam, UK) at a 1:100 dilution at 4 °C overnight. Subsequently, the sample was incubated with a biotinylated secondary antibody (Thermo Fisher, USA) for 1.5 h at room temperature. Using diaminobenzidine as an enzyme substrate for the colour reaction, haematoxylin counterstaining generated a signal. According to the order of up, down, left, right, 10 high-power fields were randomly selected to represent the number of cells in one field for subsequent statistical analyses.

### Intratumoral injection therapy of lentivirus carrying blood group antigen

Six to eight week old mice weighing approximately 20 g were first immunized with ABO blood group antigens 3 weeks before being inoculated with the cancer cell line (2 × 10^5^) and were immunized once a week after the first immunization. Five days later, they were given local injections of lentivirus-ABO drugs (1 × 10^7^/mouse, 200 μl), and the control group was the no-load virus group. Drug injections were performed on the first, third, and seventh days after treatment. In order to prevent viral drugs from entering the blood circulation, the injection process is carried out in a small amount at the edge of the tumor, and in more selected parts. When the tumour volume reached 1000 mm3, the maximum tumour diameter reached 15 mm, or tumour ulcers occurred, the mice were immediately sacrificed by cervical dislocation. After the mice were sacrificed, the tumour tissue was removed from the axilla, the tumour tissue was weighed, and the average tumour weight and standard deviation of each group of mice were calculated. At this time, the tumour volumes of the mice were measured and recorded. The tumour volume was calculated as V = Length × Width2 × 0. 50. Through the calculated tumor volume, the tumor growth curve is drawn according to the tumor growth time to judge the tumor progress. The tumor volume inhibition rate is calculated as the axillary tumor volume of each mouse minus the average tumor volume of the control group mice, and then divided by the average tumor volume of the control group mice. The mass inhibition rate is the tumor weight under the armpit of each mouse minus the average tumor weight of the control group, and then divided by the average tumor weight of the control group.

### Flow cytometry

The tumour tissue was shredded with a scalpel. The tumor chylomyces were digested in RPMI1640 + 5% FBS + 200 IU/mL type I collagenase (Thermo Scientific, USA) at 37 °C for 30 min. The tumor tissue digested into single cells was filtered with 200 mesh gauze and resuspended in 35% Percoll (GE) cell separation solution, and gently added to the upper layer of 70% Percoll cell separation solution, centripetal force 400 g, 20 min, aspirate the albuginea layer between the Percoll cell separation solution was washed once with PBS and then stained. Cell were stained with a product from Thermo Scientific (USA) to ensure that the cell number was 2 × 10^6^. Cells were stained with the following antibodies: CD45 monoclonal antibody-FITC (clone number: 30-F11) 1:100 and CD49b (integrin alpha 2) monoclonal antibody-PE (clone number: DX5), 1:100 and staining with the Fixable Viability Dye eFluor 506 was performed to eliminate dead cells. Data were collected on a BD FACS LSR II instrument with FACS Diva software (version8.0.1, BD Biosciences) and analysed using FlowJo software (version X10. 0.7r2, Tree Star Inc., Ashland, Oregon, USA). Only samples with at least 10,000 cells were included in the analysis.

### Enzyme-linked immunosorbent assay (ELISA)

The instructions of the mouse terminal complement complex C5b-9 ELISA Kit (Shanghai Enzyme Biological Company, China) were followed, and the concentration of the complement attack complex was calculated after conducting colorimetry with a microplate reader (450 nm). Then, this value was divided by the protein concentration of the corresponding sample to obtain the content of the complement attack membrane complex per gram of tumour tissue.

### Statistical analysis

Statistical analysis was performed using GraphPad Prism software (version6.01, United States). The tumour volume, CCK-8, ELISA, RT-qPCR and flow cytometry data were analysed by using an non-parametric Mann–Whitney U test. The measured data were expressed as the mean ± SD, and *p* values of less than 0.05 were considered statistically significant.

### Ethics approval

The experimental animal welfare was conducted in accordance with the Chinese Guidelines for Animal Welfare and approved by the Laboratory Animal Ethics Committee, Zhujiang Hospital of Southern Medical University.

### Consent for publication

No conflict of interest exits in the submission of this manuscript, and manuscript is approved by all authors for publication. All the authors listed have approved the manuscript that is enclosed.

## Results

### Establishment of a mouse model of the blood group A antibody

**Figure 1:**
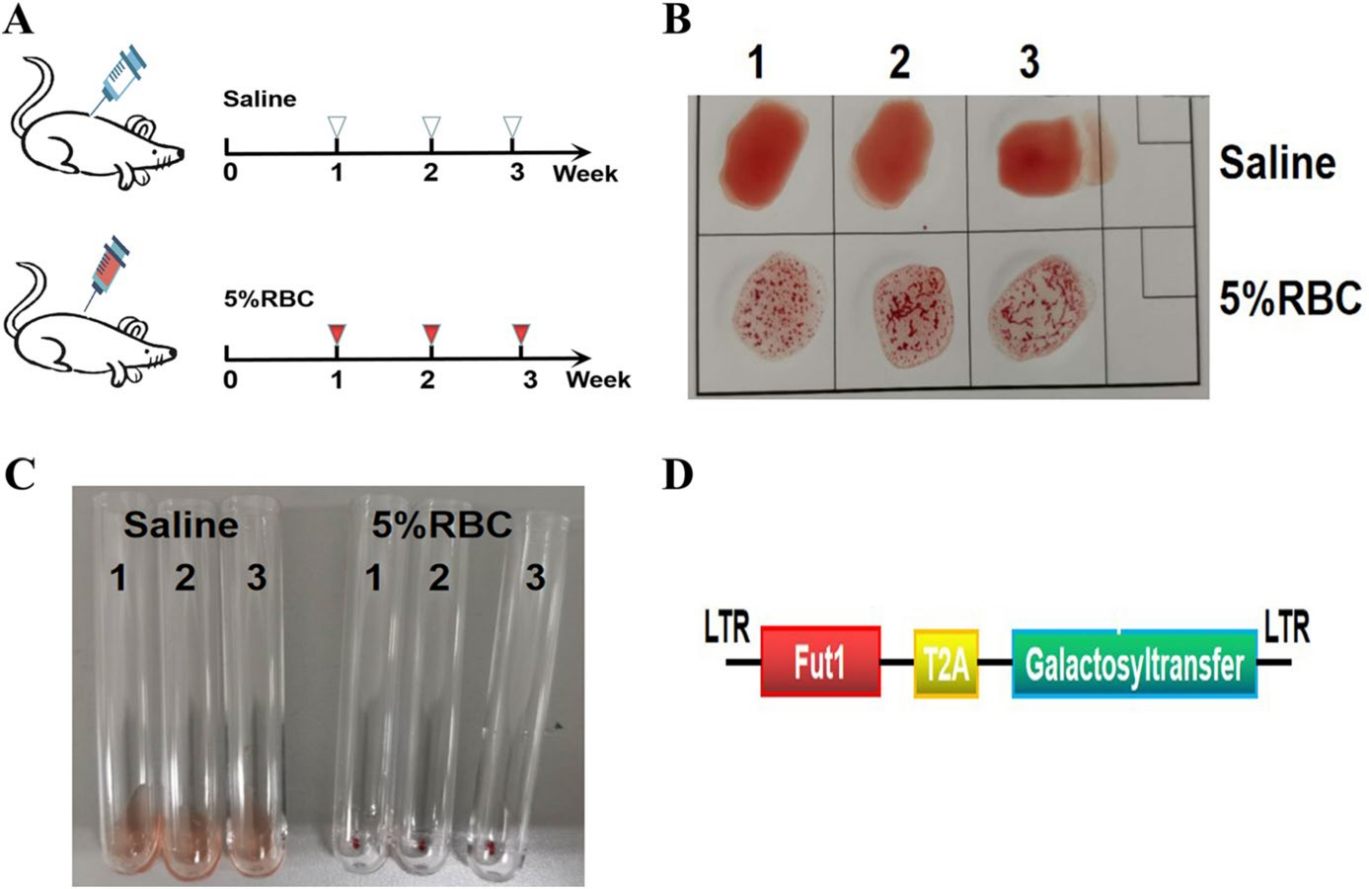
Establishment and identification of a mouse model that produces type A blood antibodies. (**A**) The flow chart of building the model. For three consecutive weeks, mice were injected with 200 μLA blood antigen into the abdominal cavity once a week, and peripheral serum was collected in the fourth week. The flow chart is drawn using the tools of WPS Office (PC version 2019, China). The slide method (**B**) and test tube method (**C**) identify blood group antibodies. The different degrees of aggregation of red blood cells indicate that the serum contains the corresponding antibodies, and the model is successfully constructed. (**D**) The schematic diagram shows the construction of a lentiviral vector containing Fut1 and the blood group gene N-acetylgalactosamine transferase.

Mice do not have ABO blood types^22^. To study the role of ABO blood group antigens in tumour treatment, we chose to use the A blood group antigen as a representative to establish a mouse model producing A blood group antibodies. (Fig. 1A) Two commonly used methods of blood type identification, the slide method (Fig. 1B) and the test tube method (Fig. 1C), proved that the immunized mice produce A blood group antibodies that agglutinates A blood group antigens, while the mice immunized with saline alone did not produce blood group A antibodies. The precursor of the ABO antigen is the H antigen. The A gene encodes a protein referred to as N-acetylgalactosamine transferase, which can convert the H antigen into the A antigen. The enzymes encoded by FUT1 are essential for the production of the oligosaccharide H antigen, and the antigen is processed and expressed on the erythrocyte membrane. Therefore, we inserted the Fut1 gene and the N-acetylgalactosamine transferase gene into the lentiviral expression vector with the expectation that the lentivirus could efficiently express the blood group A antigen on the cell membrane after the infection of tumour cells (Fig. 1D).

### Effect of the A blood group antigen on tumour formation

**Figure 2:**
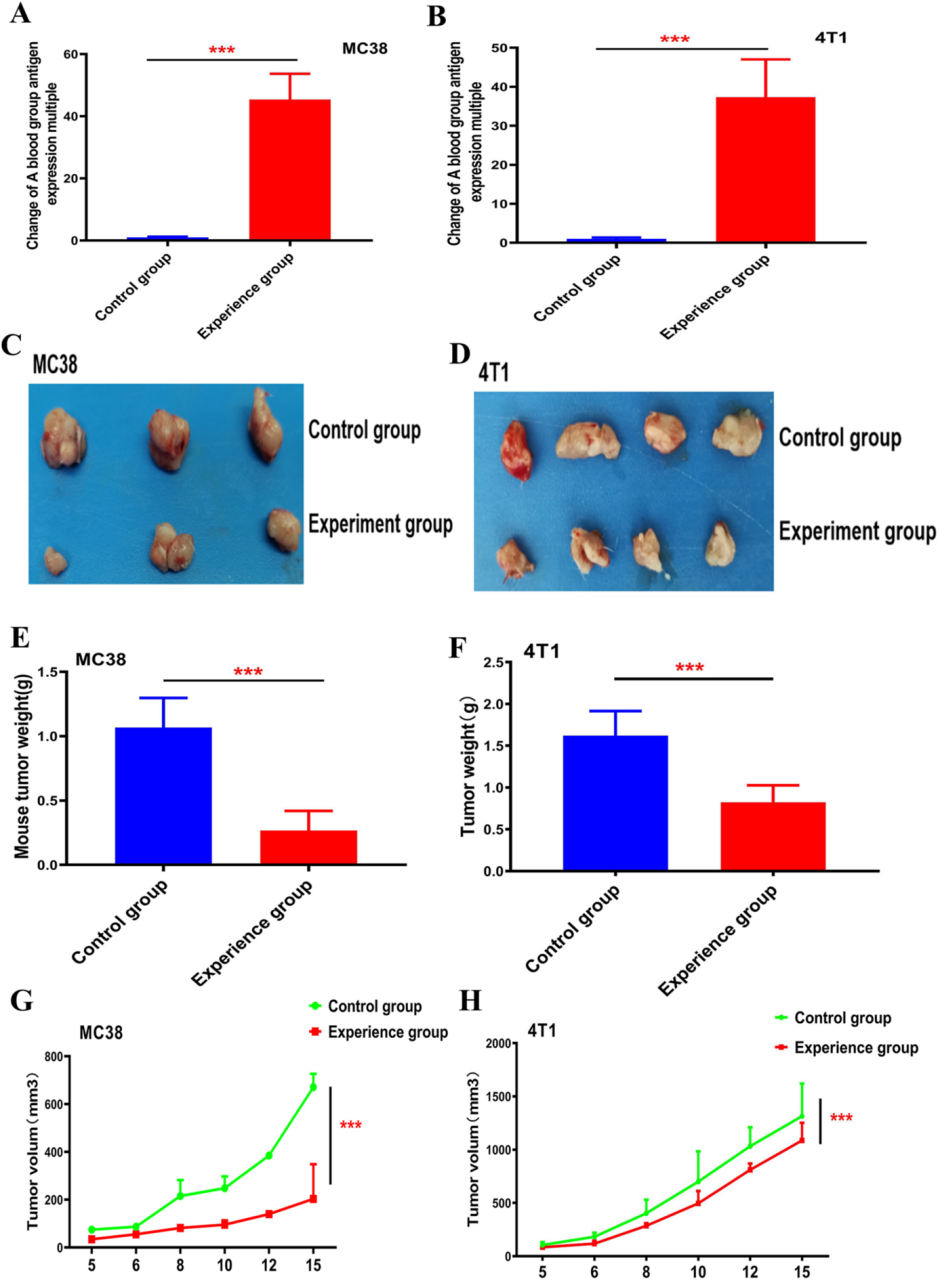
The effect of blood group A antigen on tumor formation. (**A**,**B**) Real-time PCR method was used to detect the expression level of N-acetylgalactosamine transferase gene mRNA after infection of MC38 and T41 cells with lentivirus carrying A blood group antigen, with GAPDH as the internal reference gene. Student’s t-test, mean ± SD, ***P < 0.001. (**C**,**D**) MC38 and T41 cells transfected with blood group A antigen were inoculated into the armpits of mice, and the tumors were harvested 14 days after tumor formation. (**E**,**F**) Calculate the average tumor weight and standard deviation of each group of mice, Student’s t-test, mean ± SD, ***P < 0.001. (**G**,**H**) MC38 and T41 cells infected with lentivirus carrying blood group A antigen were injected into the armpits of mice. After tumor formation, the daily tumor volume within 15 days was recorded and the tumor growth curve was drawn. The tumor volume is calculated as V = length × width^2^ × 0.50. Student’s t-test, mean ± SD, ***P < 0.001.

We chose colon cancer and breast cancer, two representative high-incidence solid tumors as the research objects. Through in vitro infection with lentivirus, we artificially overexpressed the N-acetylgalactosamine transferase gene represents blood group A antigen in the colorectal cancer cell line MC38 and the breast cancer cell line 4T1(Fig. 2A,B). To facilitate the observation of the results, we inoculated tumour cells into the axillae of the right limbs of mice capable of producing blood type A antibodies and used the empty virus in the control group. The results showed that the tumour volume was significantly smaller in the presence of A blood group antigen expression than in the control group (Fig. 2C,D). On the 15th day, the mice were sacrificed, and the tumours were weighed (Fig. 2E,F). The evaluation of the tumour volume measured each day showed that the proliferation rate of tumours with blood group A antigen expression was significantly lower than that of the control group (Fig. 2G,H). The inhibition ability of MC38 colorectal cancer tumours was stronger than that of 4T1 breast cancer tumours.

### Effect of blood group A antibodies on tumour cell growth

**Figure 3:**
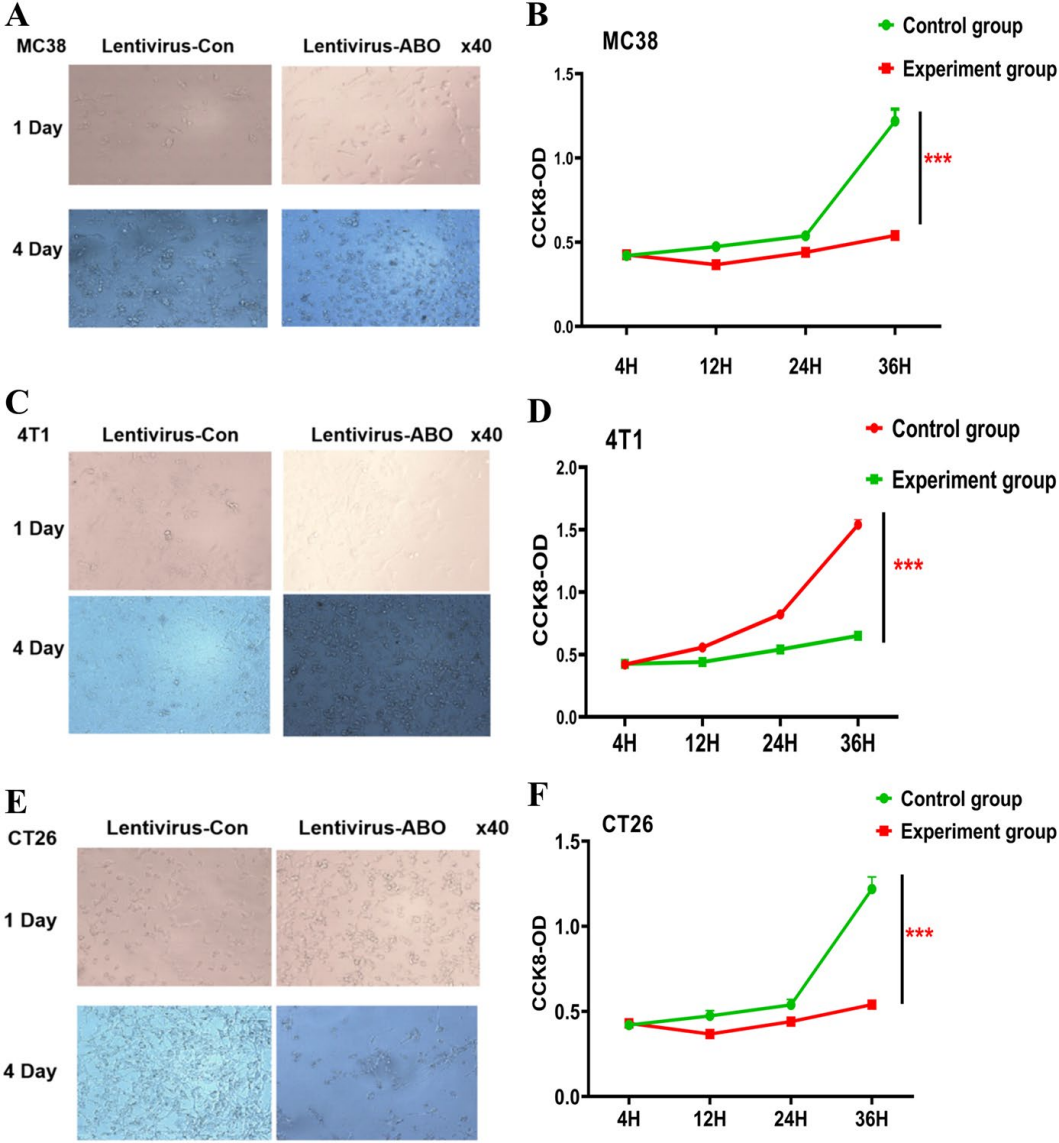
The effect of blood type A antibody on tumor cell growth. (**A**,**C**,**E**) In the medium containing blood type A antibodies, observe the growth status of MC38, CT26 and 4T1 cells expressing type A blood antigen on the first and fourth days. Mouse serum without ABO blood group antibodies was used as a control. (**B**,**D**,**F**) Observe the proliferation ability of MC38, CT26 and 4T1 cells expressing blood group A antigen in the medium containing blood group A antibody at different times. The above experiment was repeated 3 times independently, and the most typical experimental result was shown as the display. Student's t test, mean ± SD, ***P < 0.001.

Next, we sought to further prove that the tumour reduction observed in the above experiments was caused by the interaction of the blood group A antigen with the corresponding blood group antibody in the serum. Before the experiments involving these cultured tumour cells, they were infected with the lentivirus carrying the blood group A antigen for the expression of blood group A antigens. In the experiment, we added the CT26 colorectal cancer cell line. CT26 cells were derived from Babl/c mice, and MC38 cells were derived from C57 mice. The mouse serum used in the cell line experiments was also derived from corresponding germline mice, so that we could observe whether the experimental results in mice with different backgrounds were different. We used the visual observation method (Fig. 3A,C,E) and the classic CCK8(Fig. 3B,D,F) method to detect cell proliferation to assess the experimental results. The results proved that mouse serum containing the blood group A antibody significantly inhibited tumour growth and reduced the number of cells, which was effective for both colorectal cancer and breast cancer. The results of these experiments were independent of the immune background of mice.

### Therapeutic effect of the blood group A antigen on tumours

**Figure 4:**
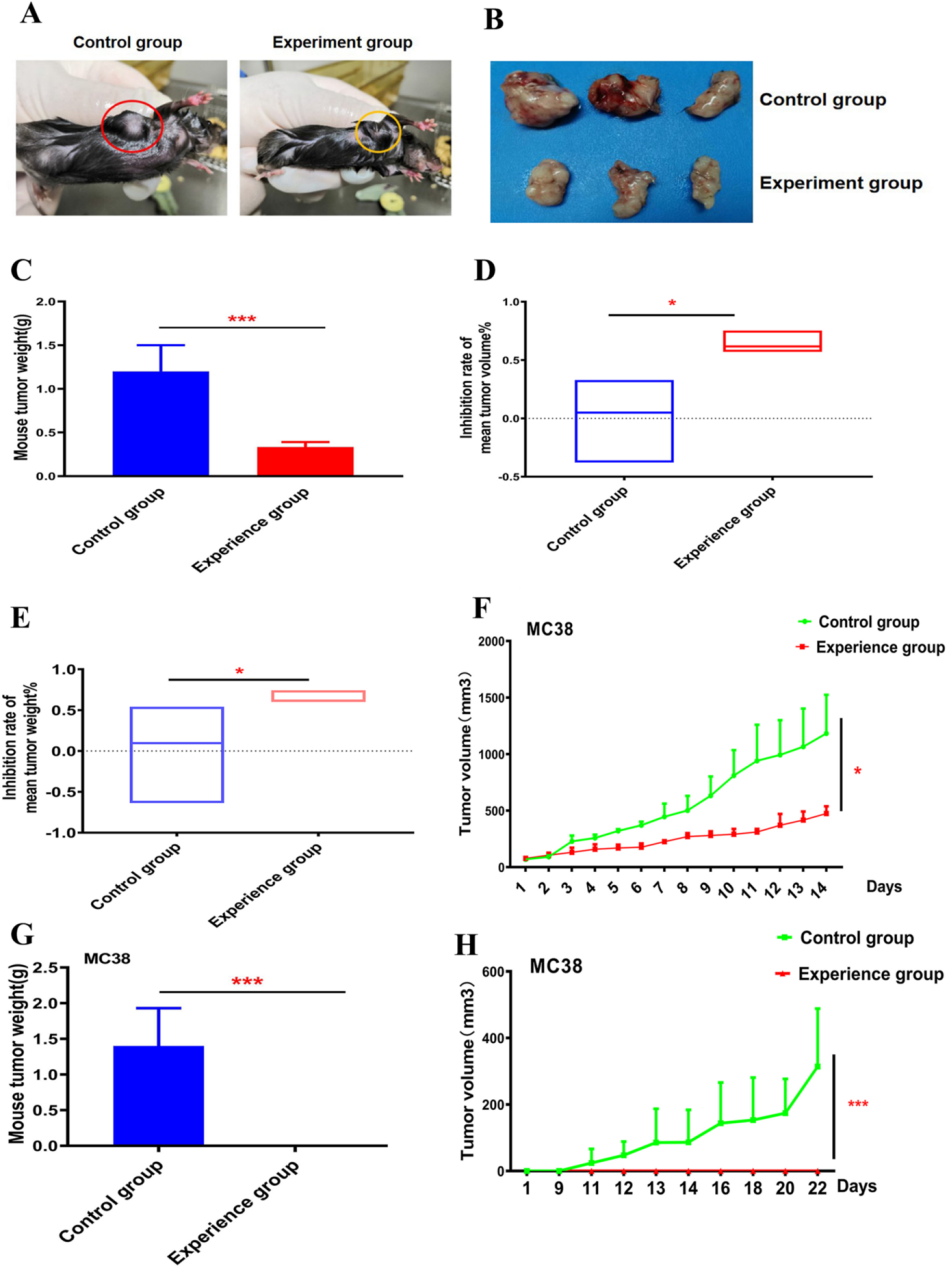
The therapeutic effect of type A blood antigen on tumor. (**A**–**F**) Five days after MC38 cell inoculation, the tumor reached a certain size and started to be injected with lentivirus carrying blood group A antigen. After 15 days, the tumor changes of the two groups of mice were compared. (**A**) Representative pictures of mouse subcutaneous tumors. (**B**) A representative picture of mouse tumors was taken. (**C**) Compare the changes in tumor weight between the two groups of mice. Student's t-test, mean ± SD, ***P < 0.001. (**D**) Inhibition of tumor volume growth by blood group A antigen, calculation formula: tumor volume of mice in the treatment group minus the average tumor volume of control mice, divided by The average tumor volume of control mice. Student's t-test, mean ± SD, *P < 0.05. (**E**) The inhibition of blood group A antigen on tumor weight, the calculation formula: the tumor weight of the mice in the treatment group minus the average weight of the tumor of the control mice, divided by the control Average weight of mouse tumors. Student’s t-test, mean ± SD, *P < 0.05. (**F**) Record tumor growth and draw growth curve. Student’s t-test, mean ± SD, *P < 0.05. One day after (**G**,**H**) MC38 cells were inoculated, the injection of lentivirus carrying blood group A antigen was started when the tumor formed. (**G**) After 15 days, compare the changes of tumor weight between the two groups of mice. Student’s t-test, mean ± SD, ***P < 0.001. (**H**) Record tumor growth and draw growth curve. Student’s t-test, mean ± SD, *P < 0.05.

We constructed mice containing antibodies to blood group A as experimental subjects, inoculated a certain number of MC38 colorectal cancer cells into the axilla, and when slightly hardened tumour tissue could be felt, intratumoural injection was performed to introduce the vector carrying the blood group A antigen by injecting it slowly. The empty lentivirus was injected in the control group. Visual inspection of the mice revealed that the tumour volume of the mice treated with the lentivirus carrying the blood group A antigen was significantly reduced (Fig. 4A). After the mice were sacrificed, the tumour tissue was removed and observed again to identify a consistent phenomenon, and the weight of the tumour was significantly reduced (Fig. 4B,C). To visually observe the degree of tumour reduction, we calculated the inhibition rate to observe the therapeutic effect of the lentivirus carrying the blood group A antigen on tumours. The experiment showed that the therapeutic effect of the lentivirus carrying the group A antigen led to more favourable results than were observed in the control group in terms of the tumour volume and weight. (Fig. 4D,E). Measuring the growth of the tumour every day proved that the lentiviral vector of the blood group antigen significantly inhibited the growth of the tumour (Fig. 4F). To further observe the therapeutic effect of the lentiviral vector of the blood type A antigen, when tumour cells were inoculated under the axilla, the inoculated site was treated by touching the small soft tissues of the mice. Surprisingly, the tumours of mice treated with the lentiviral vector of the blood group A antigen disappeared, while the tumours of the control mice grew continuously (Fig. 4G,H shows images of the collected specimen). In summary, lentiviral vectors carrying the blood type A antigen can significantly inhibit tumour growth.

### The complement system plays a role in the slowing of tumour growth by the blood group antigen

**Figure 5:**
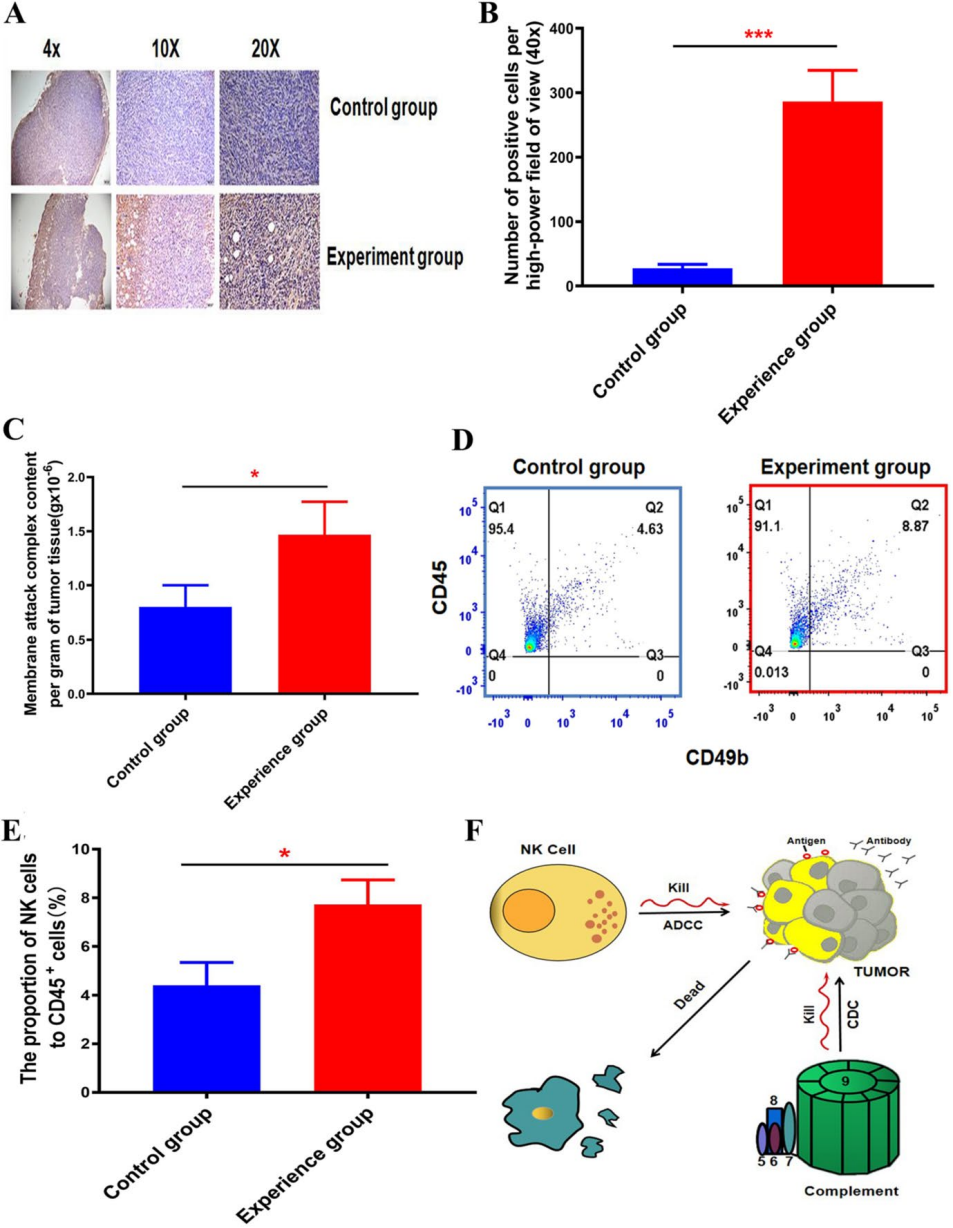
The role of the complement system in the treatment of tumors with lentiviruses carrying blood group A antigens. (**A**) Immunohistochemical method was used to detect the C5b-9 membrane attack complex in mouse tumor tissues infected with blood group antigen lentivirus. (**B**) Count the number of positive tumor cells attacked by complement in each high-power field under the microscope. Student’s t-test, mean ± SD, ***P < 0.001. (**C**) Detection of C5b-9 membrane attack compound content. After extracting total protein from the obtained tumor tissue, the protein was quantified by BAC method. ELISA method to detect the content of C5b-9 complement complex. C5b-9 complement complex content per gram of tumor tissue = C5b-9 complement complex content/total protein content. Student’s t-test, mean ± SD, *P < 0.05. (**D**–**E**) Calculate the proportion of NK cells in all immune cells using flow cytometry. Student’s t-test, mean ± SD, *P < 0.05. (**F**) The mechanism diagram of ABO blood group antigen treatment of tumors. It was drawn using WPS Office (2019 PC version, China) tools.

The immunohistochemical results showed that the number of tumour cells attacked by the membrane attack complex of complement in the tumour tissue of mice treated with the blood group A antigen was increased significantly (Fig. 5A). Quantitative analysis of the tissue sections showed that the average number of tumour cells attacked by the complement complex per high-power field in the section also increased significantly (Fig. 5B). In addition, we tested the contents of the complement complexes in tumour tissues and found that the contents of the complement attack complexes in tumour tissues after treatment with lentivirus carrying the blood group A antigen were significantly increased (Fig. 5C). Then, we used flow cytometry and found that the proportion of Natural Killer Cells (NK cells) in the tumour tissue after treatment with the lentivirus carrying the blood group A antigen was significantly increased (Fig. 5D,E). Thus may have been achieved by the activation of complement produced by the complement system to directly lyse the tumour cells and NK cells to produce ADCC (Fig. 5F).

## Discussion

In this study, we show that lentiviral vectors are very efficient for the transfer of genes in breast or colon cancer-derived cells, in vitro and in vivo. The immune response induced by the expression of blood group antigens leads to cancer cell proliferation and inhibition of tumor progression. This information will be useful for the development of novel gene therapy approaches for solid tumor treatment.

In 2005, an engineered adenovirus H101 variant (Oncorine; Shanghai Sunway Biotechnology Co, Ltd., Shanghai, China) was approved for the treatment of nasopharyngeal cancer in combined chemotherapy in China, making it the world’s first approved oncolytic virus^23^. The successful development of the drugs mentioned above greatly encouraged our research. There are a variety of viral vectors based on adenovirus, alpha virus, herpes simplex virus, coxsackie virus and vaccinia virus for the immunization studies in the experimental model. Lentiviral vectors are one of the most powerful types of gene transfer vectors and are widely used for therapeutic purposes and basic biological research^24^. The drug Glybera delivers a functional gene copy to the patient's muscle cells via a viral vector to treat lipoprotein lipase deficienc^25^. The lentiviral vector carrying the therapeutic gene is locally injected into the pancreatic cancer tissue for the treatment of pancreatic cancer to achieve ideal experimental results^26^. We showed in this study that lentiviral vectors carrying ABO blood group antigens successfully inhibited tumor growth in breast and colon cancer by causing the body's immune response.

The ABO blood group system has been identified in many primates^27^. Fan and colleagues demonstrated that there is no ABO blood group system in mice, their work laid the foundation for our experiments using mice as animal models^22^. We successfully designed a lentiviral vector carrying the Fut1 gene and N-acetylgalactosamine transferase gene according to their method, and established a mouse model containing blood type A antibodies. in vivo, tumor progression stopped after gene transfer within the tumor. while in vitro, we found that the immune response induced by blood group antigens significantly inhibited the proliferation of breast cancer cells and colon cancer cells. Here, we provide evidence, for the first time, of the notable therapeutic efficacy of lentiviral delivered therapeutic genes for established tumors. Interestingly, our research shows that this strategy is more effective for colorectal cancer than breast cancer. This may be related to the different expression levels of FUT1 or N-acetylgalactosamine transferase in different tissues. Some tissues themselves contain blood group antigens. Fan and colleagues showed that H-transferase expression was detected in colon and relatively weak expression was also found in liver, spleen, thymus, and skin. Our study showd that the treatment effect on the first day after tumor inoculation was better than that on the fifth day, which was more significant when the tumor was small. The distribution of lentiviral vectors was more even when the tumor was smaller. This may also be one of the reasons why the treatment of colorectal cancer was more effective. The antitumour efficacy of oncolytic virotherapy depends on the density and distribution of the centre of virus infection in the tumour^28^. The tissue density and structure of different tumours are different.

Next, we discussed the mechanism of blood group antigen's anti-tumor effect. The immune-mediated destruction of red blood cells is caused by two different mechanisms: one is the destruction of red blood cells by complement lysis, usually caused by antibodies^29^. When an antibody binds to a antigen, the configuration of the antibody changes, exposing the complement binding site^30^. The second mechanism is the destruction of red blood cells by immune cells through antibody-dependent cell-mediated cytotoxicity (ADCC). ADCC is mainly mediated by fine NK cells, which can recognize IgG/IgM and complement bound to red blood cells. In the process of antibody-mediated ADCC, antibodies can only specifically bind to the corresponding epitopes on target cells, and NK cells can non-specifically kill any target cells that have bound to the antibody^30^. In some immune or nonimmune haemolytic anaemia cases, the activation of complement can also mediate the destruction of red blood cells^31^. In some immune or nonimmune haemolytic anaemia cases, the activation of complement can also mediate the destruction of red blood cells ^32^.When some autoimmune diseases occur, for example, the content of complement in the peripheral blood and tissues of patients with systemic lupus erythematosus increases significantly^33^. Similar to red blood cell lysis, there are three main ways to kill tumour cells-coated with IgG antibodies: (1) receptor-mediated cytotoxicity; (2) antibody-dependent cell-mediated cytotoxicity; (3) when complement is activated by antibody-like clustered Fcγ regions, complement-mediated cytotoxicity may occur^34^. Therefore, complement has become a powerful tool for killing tumour cells. Our study also showed a significant increase in the content of C5b-9, a tumour cell complement membrane attack complex with A antigen expression, especially around the injection site, demonstrating that complement plays a key role in fighting tumours. IgG antibodies can mediate the role of these cells in ADCC, and NK cells are the main cells involved in ADCC. Activated NK cells release perforin, granzyme and other cytotoxic substances to kill target tumour cells^35^. More and more evidences show that NK cells can directly kill tumor cells without pre-sensitization, and can promote the anti-tumor effect of adaptive immunity by secreting cytokines^35,36^. Our project proved that with the expression of tumor cell A antigen, the proportion of NK cells in tumor tissues increased significantly, proving that NK cells play a key anti-tumor effect.

Tumor cells are immunoedited to make them lose immunogenicity to avoid detection by the immune system is the main way to obtain drug resistance^37^. Local injection of lentiviral vectors is used to drive solid tumors to express blood group antigens to induce immune responses. This treatment strategy effectively avoids the lack of immunogenicity caused by tumor immunoediting. Unlike some traditional immune antibody drugs or targeted drugs, in theory. This treatment strategy does not easily lead to tumor resistance to treatment. At the same time, our research group expects that simple intratumoural injections can reduce tumours and reduce the pain and cost of treatment for cancer patients.

There are also some findings of this research that should be further explored. For example, (1) how can the efficiency of the intratumoural injection of infected tumour cells and the residence time of lentiviral vectors at the tumour site be increased? (2) Since patients with the AB blood group exhibit there are no antibodies to blood groups A and B, how should cancer be treated in this group? (3) Can this approach be combined with other immune factors to increase the therapeutic effect of blood group antigens? (4) In addition to the solid tumours that we observed, the effect of this treatment method on other solid tumours remains to be studied. (5) The human ABO blood group antibody is IgM. The antibody produced by the mouse in this experiment is IgG. Theoretically, the ability of IgM antibodies to activate complement and produce ADCC is stronger than IgG. So is the effect of anti-tumor treatment in patients better than our test results? (6) Although the method of topical administration presents a clear margin of safety over systemic administration and causes less toxicity and fewer side effects, the injected dose still needs to be further explored. It is necessary to ensure the full dispersion of the drug in the tumour tissue and minimize spillage from the tumour tissue. In short, our treatment hypothesis regarding the use of blood group antigens to treat tumours was correct, and the method is effective. We hope that other researchers will consider the positive effects of blood groups on tumour treatment.
